# Molecular detection of respiratory pathogens among children aged younger than 5 years hospitalized with febrile acute respiratory infections: A prospective hospital‐based observational study in Niamey, Niger

**DOI:** 10.1002/hsr2.137

**Published:** 2019-10-11

**Authors:** Adamou Lagare, Sani Ousmane, Ibrahim Dan Dano, Bassira Issaka, Idi Issa, Halima Boubacar Mainassara, Jean Testa, Stefano Tempia, Saidou Mamadou

**Affiliations:** ^1^ Bacteriology‐Virology Unit Centre de Recherche Médicale et Sanitaire (CERMES) Niamey Niger; ^2^ Influenza Division Centers for Disease Control and Prevention Atlanta Georgia; ^3^ Influenza Program Centers for Disease Control and Prevention Pretoria South Africa; ^4^ MassGenics Duluth Duluth Georgia; ^5^ Faculté des Sciences de la Santé Université Abdou Moumouni Niamey Niger

**Keywords:** bacteria, children, febrile acute respiratory infection, Niger, viruses

## Abstract

**Background and Aims:**

In Niger, acute respiratory infections (ARIs) are the second most common cause of death in children aged younger than 5 years. However, the etiology of ARI is poorly understood in the country. This study aims to describe viral and bacterial infections among children aged younger than 5 years hospitalized with febrile ARI at two hospitals in Niamey, Niger's capital city, and the reported clinical procedures.

**Methods:**

We conducted a prospective study among children aged younger than 5 years hospitalized with febrile ARI at two national hospitals in Niamey between January and December 2015. Clinical presentation and procedures during admission were documented using a standardized case investigation form. Nasopharyngeal specimens collected from each patient were tested for a panel of respiratory viruses and bacteria using the Fast Track Diagnostic 21 Plus kit.

**Results:**

We enrolled and tested 638 children aged younger than 5 years, of whom 411 (64.4%) were aged younger than 1 year, and 15 (2.4%) died during the study period. Overall, 496/638 (77.7%) specimens tested positive for at least one respiratory virus or bacterium; of these, 195 (39.3%) tested positive for respiratory viruses, 126 (25.4%) tested positive for respiratory bacteria, and 175 (35.3%) tested positive for both respiratory viruses and bacteria. The predominant viruses detected were respiratory syncytial virus (RSV) (149/638; 23.3%), human parainfluenza virus (HPIV) types 1 to 4 (78/638; 12.2%), human rhinovirus (HRV) (62/638; 9.4%), human adenovirus (HAV) (60/638; 9.4%), and influenza virus (INF) (52/638; 8.1%). *Streptococcus pneumoniae* (249/638; 39.0%) was the most frequently detected bacterium, followed by *Staphylococcus aureus* (112/638; 12.2%) and *Haemophilus influenzae* type B (16/638; 2.5%). Chest X‐rays were performed at the discretion of the attending physician on 301 (47.2%) case patients. Of these patients, 231 (76.7%) had abnormal radiological findings. A total of 135/638 (21.2%) and 572/638 (89.7%) children received antibiotic treatment prior to admission and during admission, respectively.

**Conclusion:**

A high proportion of respiratory viruses was detected among children aged younger than 5 years with febrile ARI, raising concerns about excessive use of antibiotics in Niger.

## 1 INTRODUCTION

Acute respiratory infections (ARIs) are responsible for elevated childhood morbidity and mortality globally,^1^, ^2^ accounting for approximately four million deaths among children aged younger than 5 years in 2010 and resulting in substantial burden of healthcare systems.^3^, ^4^ In developing countries, ARIs account for 19% of all deaths among children aged younger than 5 years and 8.2% of all disabilities and premature deaths.^5^, ^6^ Therefore, data on the epidemiology and seasonality of ARI are important to develop control and prevention strategies.^7^

In Niger, the estimated mortality rate among children aged younger than 5 years was 114 per 1000 in 2012 (UNICEF child mortality estimate), and more than 80% of these deaths were associated with malaria, respiratory infections, and diarrhea. According to the 2012 Niger health statistics yearbook, the estimated case fatality proportion associated with respiratory infections was 8.7%.

In the absence of laboratory diagnosis, it is difficult to clinically differentiate between ARI‐related causative pathogens, due to similarities of symptoms.^8^, ^9^ The main causative agents of ARI are viruses and bacteria.^10^ Major viruses include respiratory syncytial virus (RSV), human rhinovirus (HRV), influenza virus (INF) types A and B, human adenovirus (HAV), human parainfluenza virus (HPIV), human metapneumovirus (HMPV), and human coronavirus (HCOV), while major bacterial pathogens include *Streptococcus pneumoniae*, *Staphylococcus aureus,* and *Haemophilus influenzae.* 11, 12, 13

In Niger, there is scarcity of data on ARIs, although a routine influenza surveillance system exists. INF has been detected in 11% of samples from children aged younger than 5 years hospitalized with severe acute respiratory illness (SARI).^14^ Knowledge on the etiology of ARI in Niger could inform the selection of other important pathogens to be included in routine surveillance and improve the clinical management of patients.

This study aims to describe the viral and bacterial infections among children aged younger than 5 years hospitalized with febrile ARI at two national hospitals of Niamey, the capital city of Niger, and the reported clinical procedures.

## 2 METHODS

### 2.1 Study design and setting

We conducted a prospective study among children aged younger than 5 years hospitalized with febrile ARI between January and December 2015. Niger has four distinct seasons as categorized by the national directorate of meteorology: the cold season (mid‐December to mid‐February), the dry season (mid‐February to May), the rainy season (June to September), and the hot season (October to mid‐December).^15^

This study was part of the “TOTAL Niger Infection Respiratoire Aiguë” (TONIRA) Project, which was funded by the TOTAL Corporate Foundation in order to strengthen medical care of febrile ARI among children aged younger than 5 years. The study was conducted at the pediatric wards of two national tertiary hospitals situated in Niamey, namely, the Hôpital National de Niamey (HNN) and the Hôpital National Lamordé (HNL). These two hospitals provide general health care to the population of Niamey estimated at 1.1 million in 2015, as well as to patients referred from across the country. Laboratory detection of respiratory viruses and bacteria were conducted at the Centre de Recherche Médicale et Sanitaire (CERMES), Niamey, Niger (the National Reference Laboratory for influenza).

Demographic and clinical data, as well as the use of antibiotics before and during admission, were collected using standard data collection forms. Malaria testing was implemented on site using thick blood film microscopy. Chest X‐rays were performed at the discretion of the attending physician, and typical abnormal radiological findings assessed included pulmonary opacities, acute bronchiolitis, and enlargement of intercostal spaces. Malnutrition was determined using the ratio of weight for height, and its level was classified as moderate or severe by the attending physician using national standards based on the 2010 Technical Guidelines for Integrated Disease Surveillance and Response in the African Region (Retrieved from https://www.afro.who.int/sites/default/files/2017-06/IDSR-Technical-Guidelines_Final_2010_0.pdf ). Febrile ARI in conjunction with malaria, malnutrition, and diarrhea were assessed during admission using a standardized case investigation form. In‐hospital outcome (ie., discharge, referral, or death) was recorded for all enrolled patients.

### 2.2 Case definition

A febrile ARI case was defined as a hospitalized child aged younger than 5 years with onset of fever 38°C or higher and cough within 10 days prior to admission and at least one of the following signs: inability to drink or breastfeed, lethargy, vomiting, convulsions, nasal flaring, chest indrawing, stridor in a calm child, or tachypnea.

### 2.3 Sampling and laboratory testing

Nasopharyngeal swabs were collected from all enrolled patients, placed in universal transport medium, stored at 4 to 8°C, and transported to CERMES within 72 hours of collection, for testing. Respiratory samples were tested for INF types A and B; HPIV types 1 to 4; HCoV NL63, 229E, OC43, and HKU1; HMPV A and B; RSV A and B; human parechovirus (HPV); human enterovirus (HEV); HAV; human bocavirus (HBoV); HRV; *S pneumoniae*; *H influenzae* type B (Hib); *S aureus*; *Mycoplasma pneumoniae*; and *Chlamydia pneumoniae* using the RT‐qPCR Fast Track Diagnostic Respiratory pathogens 21 Plus kit from Bio‐Mérieux (catalog no. FTD 2+‐96/12), following the manufacturer's instructions. An internal control provided as part of the kit was used to monitor the sample extraction and reverse transcription. RT‐qPCR was performed using the FTD Respiratory pathogens 21 plus kit as follows: the reaction volume for each test was 25 μL, consisting of 10 μL of nucleic acid and 15 μL of buffer/enzyme mix from the AgPath‐ID One‐Step RT‐PCR kit (Ambion, Life Technologies, USA, Catalog number 4387424). Amplification was performed using ABI 7500 Fast real‐time PCR system thermocycler (Applied Biosystems, USA,) and the following cycling conditions were used: 50°C at 15 minutes for reverse transcription, 95°C at 10 minutes for enzyme activation, and 40 cycles of 95°C at 8 seconds and 60°C at 34 seconds for amplification. The fluorescence was assessed at the amplification step. The positive and negative virus plasmid controls provided in the kit were included in all runs to monitor assay performance.^16^

### 2.4 Statistical analysis

Viral and/or bacterial detection was reported as percentage positive, overall and within selected categories (eg, age groups and seasons). Children aged younger than 5 years were stratified into two age groups: infants (aged younger than 1 year) and young children (aged 1‐4 years), for comparability with other studies from Africa.^17^, ^18^, ^19^, ^20^ In addition, the majority of enrolled children were aged younger than 1 year hindering our ability to categorize age in smaller age groups. Stata version 14.2 (StataCorp, College Station, Texas, USA) was used for the analysis.

### 2.5 Ethical approval

The protocol was approved by the National Ethical Consultative Committee (CCNE) of Niger and by the Clinical Research Committee (CoRC) of the Institut Pasteur, Paris. Proxy informed consent was obtained from parents or legal guardians of children. All children who did not meet the case definition or for whom verbal consent was not obtained were not included in the study.

## 3 RESULTS

### 3.1 Study population

Between January and December 2015, we enrolled and tested 638 children aged younger than 5 years. Of these, 411 (64.4%) were aged younger than 1 year, 347 (54.4%) were male, and 365 (57.3%) were admitted at the HNN. Of the comorbidities investigated, malaria, malnutrition, and diarrhea were diagnosed in 100 (15.7%), 114 (17.9%), and 114 (17.9%) case patients, respectively. Of the 114 malnourished children identified, 74 (64.9%) had severe malnutrition (Table 1). Chest X‐rays were performed on 301 (47.2%) case patients. Of these patients, 231 (76.7%) had abnormal radiological findings, of which the most common were pulmonary opacity (106; 45.9%), extension of the intercostal space (99; 42.8%), and bronchiolitis (21; 9.1%). One hundred thirty‐five (21.2%) children received antibiotic treatment within 3 days prior to admission, and 572 (89.7%) children were prescribed antibiotic treatment during admission. The in‐hospital case fatality proportion was 2.4% (15/638) overall; 2.9% (12/411) and 1.3% (3/227) among children aged younger than 1 and 1 to 4 years, respectively.

**Table 1:** Detection of respiratory viruses and bacteria among children aged <5 years hospitalized with febrile acute respiratory infection in Niamey, Niger, 2015

Characteristics	Population N (%)	Detection n (%)			
		Any virus or bacterium	Viruses only	Bacteria only	Viruses and bacteria
Total	638	496 (77.7)	195 (30.6)	126 (19.7)	175 (27.4)
Age (in y)					
<1	411 (64.4)	327 (79.6)	141 (34.3)	70 (17.0)	116 (28.2)
1‐4	227 (35.6)	169 (74.4)	54 (23.8)	56 (24.7)	59 (26.0)
Sex					
Female	291 (45.6)	231 (79.4)	86 (29.5)	61 (21.0)	84 (28.9)
Male	347 (54.4)	265 (76.4)	109 (31.4)	65 (18.7)	91 (26.2)
Season of the year^a^					
Cold	108 (84.3)	64 (59.3)	27 (25.0)	25 (23.1)	12 (11.1)
Dry	213 (33.4)	175 (82.2)	80 (37.6)	28 (13.1)	67 (31.5)
Rainy	191 (29.9)	160 (83.8)	40 (20.9)	57 (29.8)	63 (33.0)
Hot	126 (19.8)	97 (77.0)	48 (38.1)	16 (12.7)	33 (26.2)
Malaria					
No	538 (84.3)	415 (77.1)	159 (29.6)	110 (20.4)	146 (27.1)
Yes	100 (15.7)	81 (81.0)	36 (36.0)	16 (16.0)	29 (29.0)
Diarrhea					
No	524 (82.1)	407 (77.7)	164 (31.3)	100 (19.1)	143 (27.3)
Yes	114 (17.9)	89 (78.1)	31 (27.2)	26 (22.8)	32 (28.1)
Malnutrition					
No	524 (82.1)	406 (77.5)	154 (29.4)	102 (19.5)	150 (28.6)
Moderate	40 (6.3)	34 (85.0)	17 (42.5)	5 (12.5)	12 (30.0)
Severe	74 (11.6)	56 (75.7)	24 (32.4)	19 (25.7)	13 (17.6)
Antibiotics on admission					
No	66 (10.3)	50 (75.8)	20 (30.3)	9 (13.6)	21 (31.8)
Yes	572 (89.7)	446 (78.0)	175 (30.6)	117 (20.4)	154 (26.9)
Death					
No	623 (97.6)	482 (77.4)	188 (30.2)	122 (19.6)	172 (27.6)
Yes	15 (2.4)	14 (93.3)	7 (46.7)	4 (26.7)	3 (20.0)

a When the patient was hospitalized.

### 3.2 Detection of viral and bacterial agents

**Figure 1:**
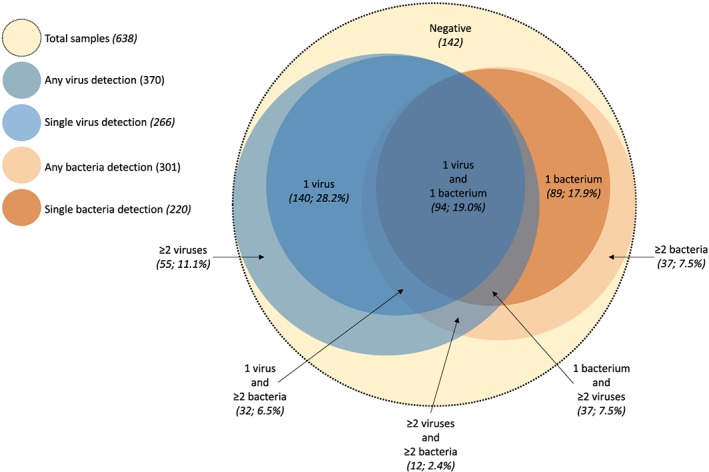
Detection (including codetection) of respiratory viruses and bacteria among children aged <5 years hospitalized with febrile acute respiratory infection in Niamey, Niger, 2015 (the size of the circles is proportional to the number of specimens within each category; percentages are expressed over total number of positive specimens: 496)

Overall, 496/638 (77.7%) specimens tested positive for at least one respiratory virus or bacterium (Table 1). Of the 496 positive specimens, 195 (39.3%) tested positive for respiratory viruses only, 126 (25.4%) tested positive for respiratory bacteria only, and 175 (35.3%) tested positive for both respiratory viruses and bacteria. Single virus or bacterium was detected in 229/496 (46.2%) positive specimens, of which 140 (61.1%) were single viral infections (Figure 1). The number of viral and/or bacterial detection among positive specimens ranged between 1 and 5.

Antibiotics were prescribed to 89.7% (175/195), 92.8% (117/126), and 88.0% (154/175) of patients with viral only, bacterial only, or viral and bacterial detection, respectively.

### 3.3 Individual detection of respiratory viruses

Overall, respiratory viruses were detected in 370/638 (58.0%) specimens. RSV (149/638; 23.3%), HPIV types 1 to 4 (78/638; 12.2%), HRV (62/638; 9.4%), HAV (60/638; 9.4%), and INF (52/638; 8.1%) were the predominant viruses detected (Table 2). Of the HPIV‐positive specimens, HPIV‐3 was the most commonly detected one (48/78; 61.5%), whereas INF type A predominated among the influenza‐positive specimens (46/52; 88.5%). HCoVs were detected in 8.0% (51/638) of specimens, and OC43 was the mostly detected one (44/51; 86.3%). HMPV, HBoV, and HEV were detected individually in less than 5% of specimens. HPV was the least commonly detected respiratory virus (1/638; 0.2%).

**Table 2:** Detection of individual respiratory viruses among children aged <5 years hospitalized with febrile acute respiratory infection in Niamey, Niger, 2015

Characteristics	Detection n (%)										
	RSV	HPIV	HRV	HAV	INF	HCoV	HMPV	HBoV	HEV	HPV	Any virus
Total (N = 638)	149 (23.3)	78 (12.2)	62 (9.7)	60 (9.4)	52 (8.1)	51 (8.0)	28 (4.4)	22 (3.4)	8 (1.2)	1 (0.2)	370 (58.0)
Age (in y)											
<1 (N = 411)	119 (28.9)	59 (14.4)	38 (9.2)	39 (9.5)	32 (7.8)	37 (9.0)	19 (4.6)	18 (4.4)	5 (1.2)	1 (0.2)	257 (62.5)
1‐4 (N = 227)	30 (13.2)	19 (8.4)	24 (10.6)	21 (9.2)	20 (8.8)	14 (6.2)	9 (4.0)	4 (1.8)	3 (1.3)	0 (0.0)	113 (49.8)
Sex											
Female (N = 291)	65 (22.3)	39 (13.4)	25 (8.6)	25 (8.6)	26 (8.9)	26 (8.9)	14 (4.8)	9 (3.9)	2 (0.7)	0 (0.0)	170 (58.4)
Male (N = 347)	84 (24.2)	39 (11.2)	37 (10.7)	35 (10.1)	26 (7.5)	25 (7.2)	14 (4.0)	13 (3.7)	6 (1.7)	1 (0.3)	200 (57.6)
Season of the year^a^											
Cold (N = 108)	12 (11.1)	4 (3.7)	3 (2.8)	4 (3.7)	16 (14.8)	4 (3.7)	2 (1.8)	4 (3.7)	1 (0.9)	0 (0.0)	39 (36.1)
Dry (N = 213)	63 (29.6)	44 (20.7)	24 (11.3)	24 (11.3)	17 (8.0)	31 (14.5)	11 (5.1)	4 (1.9)	1 (0.5)	1 (0.5)	147 (69.0)
Rainy (N = 191)	18 (9.4)	19 (9.9)	24 (12.6)	22 (11.5)	13 (6.8)	16 (8.4)	4 (2.1)	12 (6.3)	6 (3.1)	0 (0.0)	103 (53.9)
Hot (N = 126)	56 (44.4)	11 (8.7)	11 (8.7)	10 (7.9)	6 (4.8)	0 (0.0)	11 (8.7)	2 (1.6)	0 (0.0)	0 (0.0)	81 (64.3)
Malaria											
No (N = 538)	116 (21.6)	63 (11.7)	47 (8.7)	49 (9.1)	46 (8.5)	48 (8.9)	23 (4.3)	20 (3.6	6 91.1)	1 (0.2)	305 (56.7)
Yes (N = 100)	33 (33.0)	15 (15.0)	15 (15.0)	11 (11.0)	6 (6.00)	3 (3.0)	5 (5.0)	2 (2.0)	2 (2.0)	0 (0.0)	65 (65.0)
Diarrhea											
No (N = 524)	126 (24.0)	67 (12.8)	50 (9.5)	48 (9.2)	42 (8.0)	37 (7.1)	24 (4.6)	18 (3.4)	8 (1.5)	0 (0.0)	307 (58.6)
Yes (N = 114)	23 (20.2)	11 (9.6)	12 (10.5)	12 (10.5)	10 (8.8)	14 (12.3)	4 (3.5)	4 (3.5)	0 (0.0)	1 (0.9)	63 (55.3)
Malnutrition											
No (N = 524)	124 (23.7)	65 (12.4)	48 (9.2)	42 (8.1)	42 (8.0)	41 (7.8)	25 (4.8)	20 (3.8)	6 (1.1)	1 (0.2)	304 (58.0)
Moderate (N = 40)	12 (30.0)	5 (12.5)	7 (17.5)	7 (17.5)	6 (15.0)	5 (12.5)	1 (2.5)	2 (5.0)	0 (0.0)	0 (0.0)	29 (72.5)
Severe (N = 74)	13 (17.6)	8 (10.8)	7 (9.5)	11 (14.9)	4 (5.4)	5 (6.8)	2 (2.7)	0 (0.0)	2 (2.7)	0 (0.0)	37 (50.0)
Antibiotics on admission											
No (N = 66)	17 (15.8)	9 (13.6)	10 (15.1)	6 (9.1)	6 (9.1)	8 (12.1)	5 (7.6)	2 (3.0)	0 (0.0)	0 (0.0)	41 (62.1)
Yes (N = 572)	132 (23.1)	69 (12.1)	59 (9.1)	54 (9.4)	46 (8.0)	43 (7.5)	23 (4.2)	20 (3.5)	8 (1.4)	1 (0.2)	329 (57.5)
Death	1.00	.704	.002	.644	1.000	.340	1.000	1.000	1.000	1.000	.602
No (N = 623)	146 (23.4)	76 (12.2)	56 (9.0)	58 (9.3)	51 (8.2)	49 (7.9)	28 (4.5)	22 (3.5)	8 (1.3)	1 (0.2)	360 (57.8)
Yes (N = 15)	3 (20.0)	2 (13.3)	6 (40.0)	2 (13.3)	1 (6.7)	2 (13.3)	0 (0.0)	0 (0.0)	0 (0.0)	0 (0.0)	10 (66.7)
Bacteria											
Any (N = 301)	67 (22.3)	32 (10.6)	30 (10.0)	31 (10.3)	25 (8.3)	23 (7.6)	12 (4.0)	13 (4.3)	5 (1.7)	1 (0.3)	175 (58.1)
Spneu (N = 249)	50 (20.1)	28 (11.2)	24 (9.6)	28 (11.2)	20 (8.1)	18 (7.2)	11 (4.4)	10 (4.0)	3 (1.2)	1 (0.4)	142 (57.0)
Saur (N = 112)	26 (23.2)	10 (8.9)	11 (9.8)	9 (8.0)	8 (7.1)	8 (7.1)	5 (4.5)	7 (6.2)	3 (2.7)	1 (0.9)	65 (58.0)
Hib (N = 16)	2 (12.5)	3 (18.7)	2 (12.5)	2 (12.5)	4 (25.0)	0 (0.0)	0 (0.0)	0 (0.0)	0 (0.0)	0 (0.0)	8 (50.0)
Mpneu (N = 4)	0 (0.0)	0 (0.0)	1 (25.0)	1 (25.0)	1 (25.0)	0 (0.0)	0 (0.0)	0 (0.0)	0 (0.0)	0 (0.0)	2 (50.0)
Cpneu (N = 2)	1 (50.0)	0 (0.0)	0 (0.0)	0 (0.0)	0 (0.0)	1 (50.0)	0 (0.0)	0 (0.0)	0 (0.0)	0 (0.0)	2 (100.0)

Abbreviations: Cpneu, *Chlamydia pneumonia*; HAV, human andenovirus; HBoV, human bocavirus; HCoV, human coronavirus; HEV, human enterovirus; Hib, *Haemophilus influenzae* type B; HMPV, human metapneumovirus; HPV, human parechovirus; HPVI, human parainfluenza virus; HRV, human rhinovirus; INF, influenza virus; Mpneu, *Mycoplasma pneumoniae*; RSV, respiratory syncytial virus; Saur, *Staphylococcus aureus*; Spneu, *Streptococcus pneumoniae.*

a When the patient was hospitalized.

**Figure 2:**
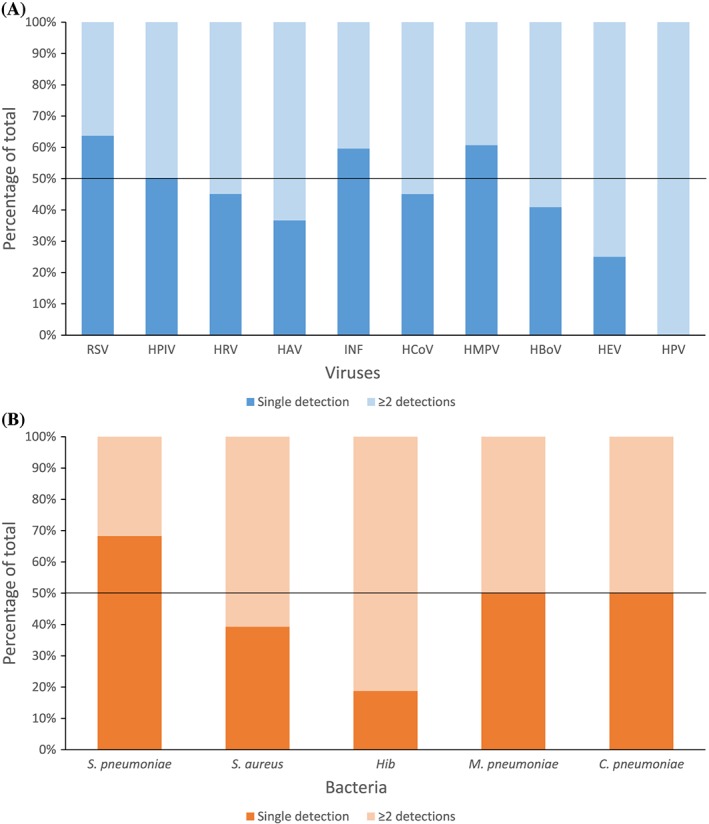
Proportion of single and multiple (A) viral and (B) bacterial detections among children aged <5 years hospitalized with febrile acute respiratory infection in Niamey, Niger, 2015. HAV, human adenovirus; HBoV, human bocavirus; HCoV, human coronavirus; HEV, human enterovirus; HPIV, human parainfluenza virus; HPV, human parechovirus; HRV, human rhinovirus; INF, influenza virus; RSV, respiratory syncytial virus

Of the specimens that tested positive for at least one respiratory virus, two or more respiratory viruses were detected in 104/370 (28.1%) specimens, of which dual detection occurred in 72/104 (69.2%) specimens. RSV (95/149; 63.8%), HMPV (17/28; 60.7%), and INF (31/52; 59.6%) were mostly detected in specimens with single viral infection, whereas HPV (1/1; 100.0%), HEV (6/8; 75.0%), HAV (38/60; 63.3%), HBoV (13/22; 59.1%), and HRV (34/62; 54.8%) were mostly detected in specimens with two or more viral infections (Figure 2A). The number of viral detections among virus‐positive specimens ranged between 1 and 4. Among patients with abnormal radiological findings, respiratory viruses were most commonly detected among patients with bronchiolitis (19/21; 90.5%). RSV and HPIV were mostly detected among children aged younger than 1 compared with 1 to 4 years (Table 2).

**Figure 3:**
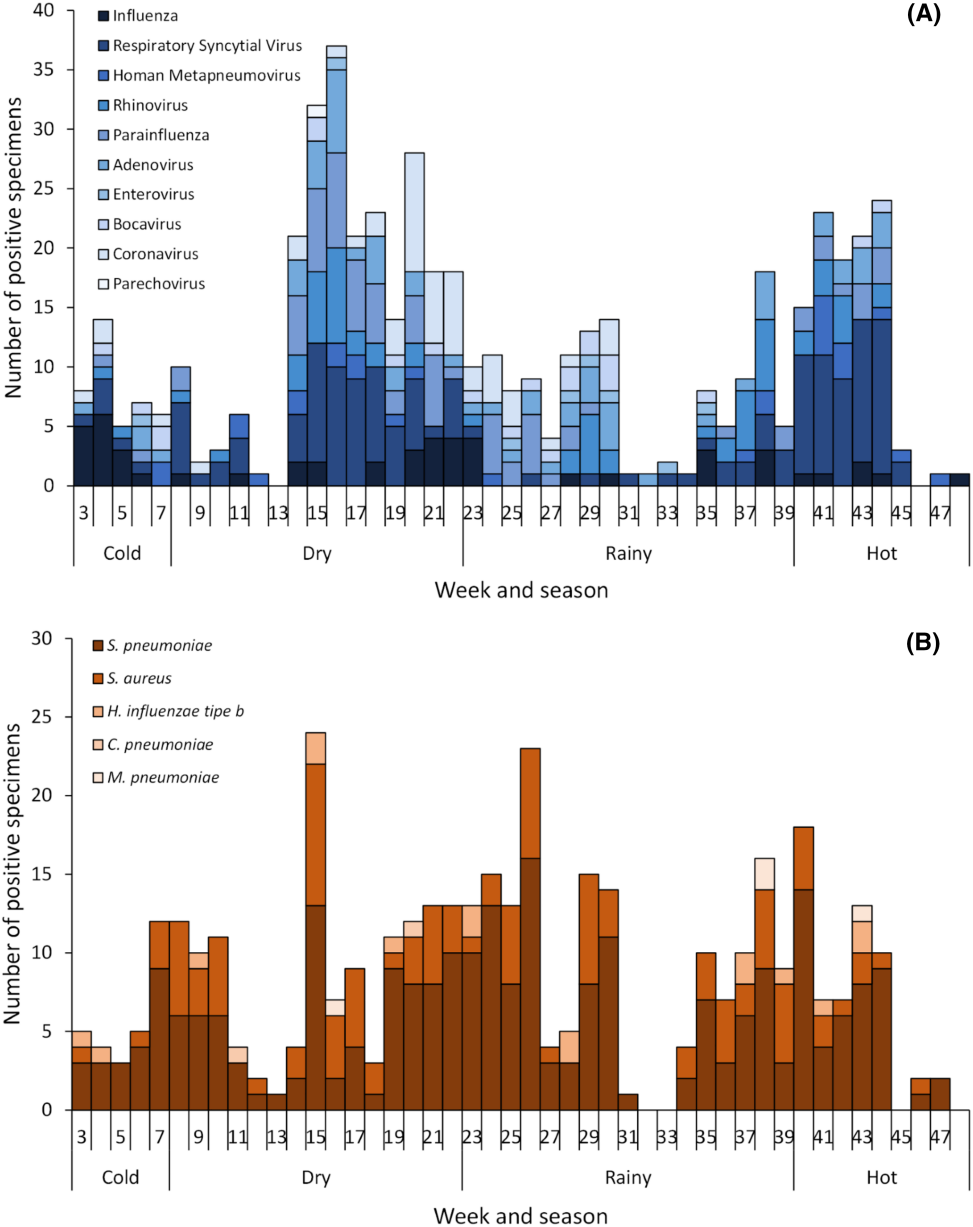
Weekly number of specimens positive for respiratory (A) viruses and (B) bacteria in Niamey, Niger, 2015

Regarding seasonality, INF was mostly detected during the cold season, HPIV and HCoV were mostly detected during the dry season, HRV was mostly detected during the rainy season, and RSV and HMPV were mostly detected during the hot season (Table 2 and Figure 3A).

### 3.4 Individual detection of respiratory bacteria

Overall, respiratory bacteria were detected in 301/638 (47.2%) specimens. *S pneumoniae* (249/638; 39.0%) was the most frequently detected bacterium, followed by *S aureus* (112/638; 12.2%) and Hib (16/638; 2.5%) (Table 3). *M pneumoniae* and *C pneumoniae* were detected individually in less than 1% of specimens.

**Table 3:** Detection of individual respiratory bacteria among children aged <5 years hospitalized with febrile acute respiratory infection in Niamey, Niger, 2015

Characteristics	Detection n (%)					
	Streptococcus pneumoniae	Staphylococcus aureus	Haemophilus influenzae type B	Mycoplasma pneumoniae	Chlamydia pneumoniae	Any bacterium
Total (N = 638)	249 (39.0)	112 (17.5)	16 (2.5)	4 (0.6)	2 (0.3)	301 (47.2)
Age (in y)						
<1 (N = 411)	152 (37.0)	68 (16.5)	8 (1.9)	3 (0.7)	1 (0.2)	186 (45.3)
1‐4 (N = 227)	97 (42.7)	44 (19.4)	8 (3.5)	1 (0.4)	1 (0.4)	115 (50.7)
Sex						
Female (N = 291)	121 (41.6)	51 (17.5)	10 (3.4)	2 (0.7)	1 (0.3)	145 (49.8)
Male (N = 347)	128 (36.9)	61 (17.6)	6 (1.7)	2 (0.6)	1 (0.3)	156 (45.0)
Season of the year^a^						
Cold (N = 108)	31 (28.7)	11 (10.2)	3 (2.8)	0 (0.0)	0 (0.0)	37 (34.3)
Dry (N = 213)	69 (32.4)	43 (20.2)	3 (1.4)	1 (0.5)	2 (0.9)	95 (44.6)
Rainy (N = 191)	105 (55.0)	47 (24.6)	7 (3.7)	2 (1.0)	0 (0.0)	120 (62.8)
Hot (N = 126)	44 (34.9)	11 (8.7)	3 (2.4)	2 (0.8)	0 (0.0)	49 (38.9)
Malaria						
No (N = 538)	210 (39.0)	93 (17.3)	14 (2.6)	2 (0.4)	2 (0.4)	256 (47.6)
Yes (N = 100)	39 (39.0)	19 (19.0)	2 (2.0)	2 (2.0)	0 (0.0)	45 (45.0)
Diarrhea						
No (N = 524)	196 (37.4)	95 (18.1)	12 (2.3)	3 (0.6)	2 (0.4)	243 (46.4)
Yes (N = 114)	53 (46.5)	17 (14.9)	4 (3.5)	1 (0.9)	0 (0.0)	58 (50.9)
Malnutrition						
No (N = 524)	208 (39.7)	95 (18.1)	14 (2.7)	4 (0.8)	2 (0.4)	252 (48.1)
Moderate (N = 40)	14 (35.0)	6 (15.0)	0 (0.0)	0 (0.0)	0 (0.0)	17 (42.5)
Severe (N = 74)	27 (36.5)	11 (14.9(	2 (2.7)	0 (0.0)	0 (0.0)	32 (43.2)
Antibiotics on admission						
No (N = 66)	21 (31.8)	15 (22.7)	0 (0.0)	0 (0.0)	1 (1.5)	30 (45.4)
Yes (N = 572)	228 (39.9)	97 (17.0)	16 (2.8)	4 (0.7)	1 (0.2)	271 (47.4)
Death						
No (N = 623)	243 (39.0)	111 (17.8)	16 (2.6)	3 (0.5)	2 (0.3)	294 (47.2)
Yes (N = 15)	6 (40.0)	1 (6.7)	0 (0.0)	1 (6.7)	0 (0.0)	7 (46.7)
Viruses						
Any (N = 370)	142 (38.4)	65 (17.6)	8 (2.2)	2 (0.5)	2 (0.3)	175 (47.3)
RSV (N = 149)	50 (33.6)	26 (17.4)	2 (1.3)	0 (0.0)	1 (0.7)	67 (45.0)
HPIV (N = 78)	28 (35.9)	10 (12.8)	3 (3.8)	0 (0.0)	0 (0.0)	32 (41.0)
HRV (N = 62)	24 (38.7)	11 (17.7)	2 (3.2)	1 (1.6)	0 (0.0)	30 (48.4)
HAV (N = 60)	28 (46.7)	9 (15.0)	2 (3.3)	1 (1.7)	0 (0.0)	31 (51.7)
INF (N = 52)	20 (38.5)	8 (15.4)	4 (7.7)	1 (1.9)	0 (0.0)	25 (48.1)
HCoV (N = 51)	18 (35.3)	8 (15.7)	0 (0.0)	0 (0.0)	1 (2.0)	23 (45.1)
HMPV (N = 28)	11 (39.0)	5 (17.9)	0 (0.0)	0 (0.0)	0 (0.0)	12 (42.9)
HBoV (N = 22)	10 (45.4)	7 (31.8)	0 (0.0)	0 (0.0)	0 (0.0)	13 (59.1)
HPV (N=1)	1 (100.0)	1 (100.0)	0 (0.0)	0 (0.0)	0 (0.0)	1 (100.0)

Abbreviations: HAV, human andenovirus; HBoV, human bocavirus; HCoV, human coronavirus; HEV, human enterovirus; HMPV, human metapneumovirus; HPV, human parechovirus. HPVI, human parainfluenza virus; HRV, human rhinovirus; INF, influenza virus; RSV, respiratory syncytial virus.

a When the patient was hospitalized.

Of the specimens that tested positive for at least one respiratory bacteria, two or more respiratory bacteria were detected in 81/220 (26.9%) specimens, of which dual detection occurred in 80/81 (98.8%) of specimens. *S pneumoniae* (170/249; 68.3%) was mostly detected in specimens with a single bacterial infection, whereas Hib (13/16; 54.8%) and *S aureus* (68/112; 60.7%) were mostly detected in specimens with two or more bacterial infections (Figure 2B). The number of bacterial detections among bacteria‐positive specimens ranged between 1 and 3. Among patients with abnormal radiological findings, respiratory bacteria were most commonly detected among patients with pulmonary opacity (87/106; 90.5%).

*S pneumoniae* and *S aureus* were mostly detected during the rainy season (June to September) (Table 3 and Figure 3B).

## 4 DISCUSSION

We report the detection of respiratory viruses and bacteria among children aged younger than 5 years hospitalized with febrile ARI in Niger between January and December 2015. At least one respiratory virus or bacteria was found in 77.7% of samples tested. This proportion was similar to that reported in a previous study on the etiology of ARI in children aged younger than 5 years in Niger (78%), although on a limited number of samples.^8^ This finding was also consistent with those of other similar studies conducted in Africa (range 55%‐70%)^21^, ^22^ and Asia (range 45%‐65%).^23^, ^24^, ^25^

The positivity proportion of respiratory viruses varied considerably. RSV, HPIV, HRV, HAV, and INF were the most commonly detected viruses. As reported in other studies, RSV, INF, and HMPV were mostly detected as single viral infections.^26^, ^27^ RSV, HRV, and HPIV were also the most predominant viruses detected in children aged younger than 5 years in a previous study conducted in Niger.^8^ RSV is considered to be a major cause of ARI in children aged younger than 5 years,^26^, ^28^, ^29^, ^30^ and it was the most commonly detected virus also in this study (149/638; 23.3%).

Among the cases in which HPIV and HCoV were detected, HPIV type 3 and HCoV type OC43 were the predominant types, and this is consistent with previous studies.^27^, ^31^ In this study, INFs were detected in 8.5% of the cases, which is in agreement with findings from the national influenza surveillance^14^ and other related studies in Africa among patients hospitalized with SARI.^18^, ^32^, ^33^ A higher influenza positivity proportion has been reported among outpatients with influenza‐like illness.^13^, ^28^

During the 1‐year study period, respiratory viruses were detected throughout the four seasons.^15^ However, RSV was mostly detected during the hot and dry seasons with higher temperature and lower relative humidity,^7^ whereas INFs were mostly detected during the cold and rainy seasons, as reported from influenza sentinel surveillance in Niger.^14^, ^34^ If these seasonal patterns are confirmed in multiyear studies, they could be useful for epidemic preparedness and response plans.

High colonization rates of *S pneumoniae*, *S aureus,* and Hib have been reported in children, but only a fraction of colonization events have been reported to result in disease.^35^, ^36^ *S pneumoniae* was detected in 39.5% of specimens, and it was the bacteria predominantly detected in our samples. However, the colonization rate found in this study was lower than that reported in a study conducted in 2014 (54.5%), which could be attributed to the introduction of the 13‐valent pneumococcal conjugate vaccination (PCV13) in Niger that same year.^37^ The very low detection rate of Hib (2.5%) may also be attributed to the introduction of the Hib conjugate (tetanus toxoid) vaccine in the expanded immunization program in 2008 in Niger.^38^ Uncommon respiratory bacterial pathogens including *M pneumoniae* and *C pneumoniae* were detected at a very low rate (less than 1%), and this correlates with the results reported in previous studies.^10^, ^26^, ^27^

In our study, 21.2% of children had already received antibiotics before hospitalization, and nearly all children (89.7%) received antibiotics during admission. These findings are consistent with a study from Mali, which reported a rate of antibiotics uptake of 39.8% before admission and elevated antibiotics usage (more than 95%) on admission.^39^ The national guidelines for ARI treatment based on the Integrated Management of Childhood Illness (Manuel sur la Prise en Charge Integrée des Maladies de l'Enfant (PCIME) (2001), retrieved from https://apps.who.int/iris/bitstream/handle/10665/67167/WHO_FCH_CAH_00.12_fre.pdf;jsessionid=65C124F019A55837AF0B229E9AB1B21A?sequence=1 ) include the systematic use of antibiotics for empiric treatment to prevent superinfection. However, considering the high number of patients from whom only respiratory viruses were detected, this practice raises concern about the potentially excessive use of antibiotics—and resistance development—in Niger.^28^

About 15% to 17% of patients with febrile ARI also had diarrhea, were malnourished, or were coinfected with malaria. This could be explained by the fact that these infections constitute some of the leading public health concerns in Niger and are responsible for elevated childhood mortality (Annuaire des statistiques sanitaires du Niger) (2016). Retrieved from http://www.stat-niger.org/statistique/file/Annuaires_Statistiques/snis/Annuaire_statistiques_2016.pdf).

Our study presents some limitations. First, the study spanned over a period of only 12 months, limiting our ability to fully assess the temporal circulation pattern of the investigated agents. Due to the small sample size, we may also have been underpowered to detect small variations in the temporal distribution of the investigated agents. Second, attribution of causality remains challenging due to the lack of controls in our study. The association of pathogen detection with illness could not be well assessed, although most of the viral and bacterial pathogens identified in this study have been described in previous case control studies as causative agents of ARI.^10^, ^39^ In addition, we did not collect blood samples to assess whether the detection of common bacterial colonizers of the nasopharynx, such as *S pneumoniae*, were associated with invasive disease. Third, we did not record the number of patients with febrile ARI that refused enrollment, hindering our ability to estimate underenrollment. Last, given that only patients with febrile ARI were enrolled, this could have resulted in an underestimation of the RSV burden since a large proportion of RSV‐related illness is not associated with fever.^40^

## 5 CONCLUSIONS

In this 1‐year prospective study, both viral and bacterial pathogens were detected in high proportion among hospitalized children aged younger than 5 years with febrile ARI in Niamey, Niger. However, further investigations should be carried out to assess risk factors and association of pathogens with illness. Although national guidelines for ARI treatment recommend systematic empiric antibiotic therapy, our results suggest that antibiotic use might be unnecessary in most cases, given the predominance of viral infections as potential cause of febrile ARI. Furthermore, other predisposing diseases such as malaria, malnutrition, and diarrhea may be contributory factors to febrile ARI exacerbation among hospitalized children.

## AUTHOR CONTRIBUTIONS

Conceptualization: Adamou Lagare, Sani Ousmane, Ibrahim Dan Dano, Halima Boubacar Mainassara, Jean Testa, Saidou Mamadou

Funding Acquisition: Adamou Lagare, Sani Ousmane, Jean Testa, Saidou Mamadou

Investigation: Adamou Lagare, Sani Ousmane, Ibrahim Dan Dano, Bassira Issaka, Idi Issa,

Halima Boubacar Mainassara, Jean Testa, Stefano Tempia, Saidou Mamadou

Project Administration: Adamou Lagare, Sani Ousmane, Jean Testa

Visualization: Adamou Lagare, Saidou Mamadou

Writing – Original Draft Preparation: Adamou Lagare, Sani Ousmane, Jean Testa, Stefano Tempia, Saidou Mamadou

Writing – Review & Editing: Adamou Lagare, Sani Ousmane, Ibrahim Dan Dano,

Bassira Issaka, Idi Issa, Halima Boubacar Mainassara, Jean Testa, Stefano Tempia, Saidou Mamadou

All authors have read and approved the final version of the manuscript.

Adamou Lagare had full access to the data and takes responsibility for the integrity of the data and the accuracy of the data analysis.

## TRANSPARENCY STATEMENT

The lead author/manuscript guarantor (Adamou Lagare) affirms that this manuscript is an honest, accurate, and transparent account of the study being reported; that no important aspects of the study have been omitted; and that any discrepancies from the study as planned (and, if relevant, registered) have been explained.

## CONFLICTS OF INTERESTS

All authors declare that they have no commercial or other associations that may pose a conflict of interest.

## DISCLAIMER

The findings and conclusions in this report are those of the authors and do not necessarily represent the official position of the US Centers for Disease Control and Prevention, USA.

## Data Availability

The authors confirm that the data supporting the findings of this study are available within the article and its supplementary materials.
